# In‐hospital and long‐term mortality for acute heart failure: analysis at the time of admission to the emergency department

**DOI:** 10.1002/ehf2.12847

**Published:** 2020-06-26

**Authors:** Carlo Lombardi, Giulia Peveri, Dario Cani, Federica Latta, Andrea Bonelli, Daniela Tomasoni, Marco Sbolli, Alice Ravera, Valentina Carubelli, Nicola Saccani, Claudia Specchia, Marco Metra

**Affiliations:** ^1^ Department of Medical and Surgical Specialties, Radiological Sciences and Public Health University University of Brescia Spedali Civili of Brescia Brescia Italy; ^2^ Department of Molecular and Translational Medicine University of Brescia Brescia Italy

**Keywords:** Acute heart failure, Mortality predictors, Ejection fraction, Creatinine, NYHA class

## Abstract

**Aims:**

Acute heart failure (AHF) leads to a drastic increase in mortality and rehospitalization. The aim of the study was to identify prognostic variables in a real‐life population of AHF patients admitted to the emergency department with acute shortness of breath.

**Methods and results:**

We evaluated potential predictors of mortality in 728 consecutive patients admitted to the emergency department with AHF. Possible predictors of all‐cause and cardiovascular (CV) mortality were investigated by Cox and Fine and Gray models at multivariable analysis. Among the 728 patients, 256 died during the entire follow‐up, 142 of these due to CV cause. The 1 year mortality rate was 20%, with the highest risk of death during the index hospitalization (with 8% estimate in‐hospital mortality at 30 days). A higher risk of events during the index hospitalization was more evident for the CV deaths, for which we found a cumulative 1 year incidence of 12% with a cumulative incidence in the first 30 days of hospitalization of about 5%. At multivariable analysis, age (*P* < 0.001), New York Heart Association (NYHA) class IV vs. I–II–III (*P* = 0.001), systolic blood pressure (*P* < 0.001), non‐cardiac co‐morbidities (≥3 vs. 0, *P* = 0.05), oxygen saturation (*P* = 0.03), serum creatinine (*P* < 0.001), and left ventricular ejection fraction (LVEF) (40–49% vs. <40%, *P* = 0.004; ≥50% vs. <40%, *P* = 0.003) were independent predictors of all‐cause mortality during the entire follow‐up. Age (*P* = 0.03), systolic blood pressure (*P* = 0.01), oxygen saturation (*P* = 0.03), serum creatinine (*P* = 0.02), and LVEF (40–49% vs. <40%, *P* = 0.03; ≥50% vs. <40%, *P* = 0.004) were independent predictors of CV mortality during the entire follow‐up. NYHA class IV vs. I–II–III (*P* < 0.001), serum creatinine (*P* = 0.01), and LVEF (40–49% vs. <40%, *P* = 0.02; ≥50% vs. <40%, *P* < 0.001) remained independent predictors for in‐hospital death, while only serum creatinine (*P* = 0.04), LVEF (40–49% vs. <40%: 0.32, *P* = 0.04; ≥50% vs. <40%, *P* < 0.001), and NYHA class vs. I–II–III (*P* = 0.02) remained predictors for in‐hospital CV mortality.

**Conclusions:**

In this real‐life cohort of patients with AHF, the results showed a similar mortality rate comparing with other analysis and with the most important registries. Age, NYHA class IV, systolic blood pressure, creatinine levels, sodium levels, and ejection fraction were independent predictors of 1 year mortality, while LVEF <40% was the only predictor of both all‐cause mortality and CV mortality.

## Introduction

Acute heart failure (AHF) is a major cause of hospitalization and is associated with a dramatic increase in mortality and morbidity (rehospitalization).^1^, ^2^ The impact on cost of care is considerable too, as the global estimate is around $108 billion per annum. In the last two decades, AHF has been widely studied to understand clinical characteristics, risk factors, and predictors of bad outcome, with the purpose of improving prevention and treatment, and to reduce length of hospitalization, costs, and mortality.^3^ Also, prognostic variables have been identified using data from registries and clinical trials. ADHERE is the most relevant AHF multicentre registry that included 107 362 hospitalizations between 1 October 2001 and 4 January 2004 from 282 different Hospitals in the USA.^4^ The in‐hospital mortality rate founded was 4%, and the 1 year mortality 36%. Similarly, 211 cardiology centres from 21 European countries contributed to create the European Society of Cardiology Heart Failure (ESC‐HF) long‐term registry.^5^ Patients were enrolled between May 2011 and April 2014, 40.5% of them after hospitalization for AHF while the others were ambulatory patients with chronic heart failure (HF). Data after 1 year follow‐up underlined that the mortality rate for patients with AHF was very higher than ambulatory HF patients (23.6% vs. 6.4%) as well as the risk of a new hospitalization.

These registries allowed to identify a lot of predictors of all‐cause mortality, and these have been largely confirmed by other analyses. However, most of these analyses were based on selected patients enrolled in a clinical trial and admitted to a cardiology department. Our single‐centre experience points out that not all patients that come to the emergency department (ED) for signs and symptoms of AHF were hospitalized in the cardiology department but also in medicine department. This could be an outstanding bias for those registries or clinical trials typically focused on AHF patients hospitalized only in the cardiology department. The aim of the study was to identify prognostic variables in a real‐life population of ACF patients admitted to the ED with acute shortness of breath.

## Methods

### Patient population

In this study, we included the patients admitted to the ED with acute dyspnoea with either de novo AHF or acute decompensated HF. From December 2014 to August 2016, 728 consecutive patients admitted to the ED of ASST Spedali Civili of Brescia were included in this analysis. The diagnosis of ACF was established at the time of admission to the ED after performing the following tests: clinical examination, blood tests, chest radiography, electrocardiogram, and echocardiogram. The diagnosis of ACF required dyspnoea at rest or minimal exercise and clinical signs of congestion/fluid overload. The diagnosis had to be confirmed by the presence of at least two of the following elements: congestion at RX, echocardiographic evidence of congestion, and elevated natriuretic peptide levels when available [brain natriuretic peptide = 250 pg/mL or N‐terminal pro‐brain natriuretic peptide (NT‐proBNP) = 1000 pg/mL]. The parameters assessed for congestion by echocardiography were as follows: inferior vena cava size and collapsibility, mitral valve inflow velocities, ratio of E to e′ (E/e′), and systolic pulmonary pressure. Patients with acute coronary syndrome, myocarditis, infections, and cardiogenic shock were excluded. All patients were evaluated in the ED by a cardiologist before admission.

The first hospitalization only was considered and defined as the index hospitalization. Possible subsequent hospitalizations were recorded as part of the patient's follow‐up. Patients were prospectively followed in accordance with the usual practice, and their vital status was ascertained until March 2019.

### Data collection

At the time of presentation in ED, the clinical evaluation included some common parameters, including age, systolic and diastolic blood pressure (SBP and DBP), heart rate, oxygen saturation, and New York Heart Association (NYHA) class.

A medical history was collected by physicians, with special regard to the cardiologic history (e.g. previous acute myocardial infarction, atrial fibrillation, and coronary artery bypass grafting).

For every patient, some laboratory measurements were evaluated including haemoglobin, haematocrit, white blood cells count, creatinine, bilirubin, sodium, potassium, aspartate transaminase, alanine transaminase, and cardiac Troponin I (TnI). Differently, NT‐proBNP such as C‐reactive protein (CRP) was not tested in every medical situation because it is not part of the analysis routine package. In the ED, every subject underwent to echocardiographic assessment of left ventricular ejection fraction (EF). Finally, we evaluated also if acute intravenous treatment with furosemide and inotropes or vasodilators or invasive procedures such as coronary revascularization or continuous venovenous haemofiltration (CVVH) were needed.

### Endpoints

We considered mortality for all causes and for cardiovascular (CV) cause as endpoints. In particular, the outcomes of interest were (i) mortality during the entire follow‐up and (ii) in‐hospital mortality.

### Statistical analysis

Possible predictors of the endpoints were investigated by univariate analysis. Categorical variables were reported as absolute frequencies and percentages. Comparison among groups was performed using the chi‐squared test for proportions. Fisher's exact test was used instead of chi‐squared test if any expected count was less than 5. Continuous variables were expressed as median and interquartile range (IQR) and compared between two independent groups by the Wilcoxon test.

The overall survival observed in the cohort was estimated using the Kaplan–Meier method. CV mortality risk was estimated in terms of cumulative incidence function, taking into account death for other causes as a competing event.

All variables clinically relevant or statistically significant at the univariate analysis were included in a multiple Cox regression model for all‐cause death outcomes, and in a multiple Fine and Gray regression model for CV death outcomes, to identify independent predictors.

The hazard ratios (HRs), 95% confidence intervals (CIs), and *P*‐values from a Wald test were reported.

If missing data for a potentially relevant variable exceeded 20% of all observation, we decided to exclude named variable from our final models. For the remaining variables, if missing data still existed, we considered a multiple imputation correction with five imputations, each obtained using the fully conditional specification method (with discriminant method for the imputation of the class variables and regression method for continuous variables). We included these investigations as sensitivity analyses.

For all models, we reported the HRs, 95% CIs, and *P*‐values from a Wald test. A two‐tailed *P*‐value <0.05 was considered statistically significant. Statistical analyses were performed using SAS statistical software version 9.4 (SAS Institute, Inc., Cary, NC, USA).

## Results

### Baseline characteristics

Our study cohort includes 728 consecutive patients hospitalized with a diagnosis of AHF between December 2014 and August 2016. Their median age was of 82 years (IQR: 75–87 years). Two hundred thirty patients (31.6%) were hospitalized in Cardiology/Cardiac Intensive Care Unit, 459 (63.0%) in Internal Medicine, and the remaining 39 (5.4%) in other institutes. The non‐cardiac co‐morbidities of the population are synthesized in Supporting Information, *Table* *S1*.

### Follow‐up

**Figure 1:**
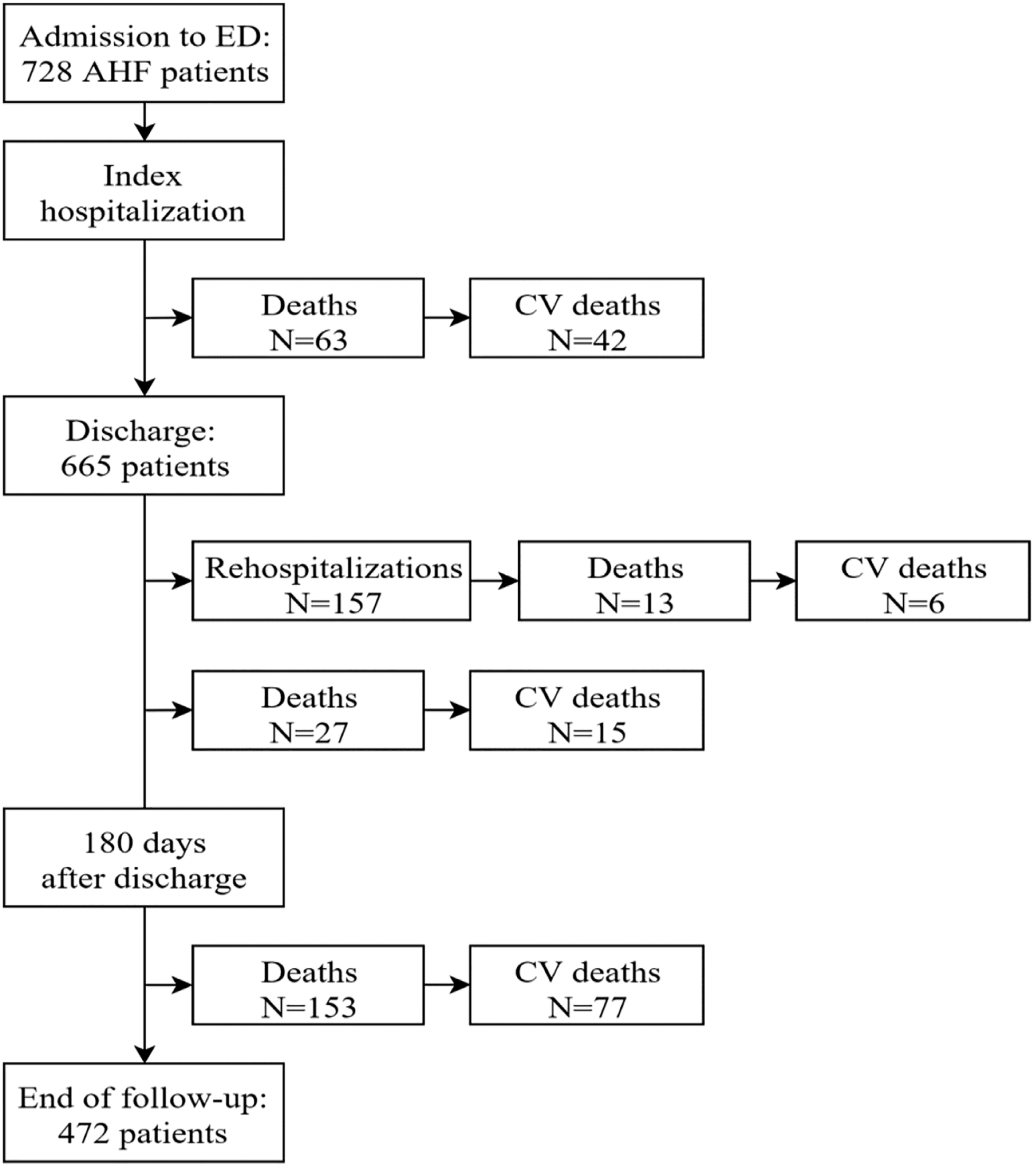
Study flow diagram: recruitment of acute heart failure (AHF) patients, follow‐up, and outcomes. CV, cardiovascular; ED, emergency department.

Flow diagram of the study population is shown in *Figure* *1*. Median follow‐up time was 35 (IQR: 19–43) months. Median length of the hospital stay was 11 days (IQR: 7–15).

During the entire follow‐up period, 256 (35.1%) patients died, of which 140 (54.7%) for CV disease. Sixty‐three patients (8.7%) died during the index hospitalization, of which 42 (66.7%) due to CV causes.

Death occurred after a median of 11 (IQR: 5–17) days for in‐hospital deaths.

**Figure 2:**
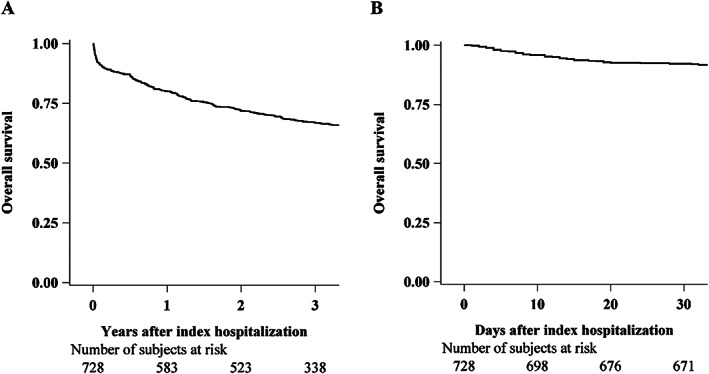
Kaplan–Meier survival curves for all‐cause death during the entire follow‐up after index hospitalization (A) and during index hospitalization time (B).

**Figure 3:**
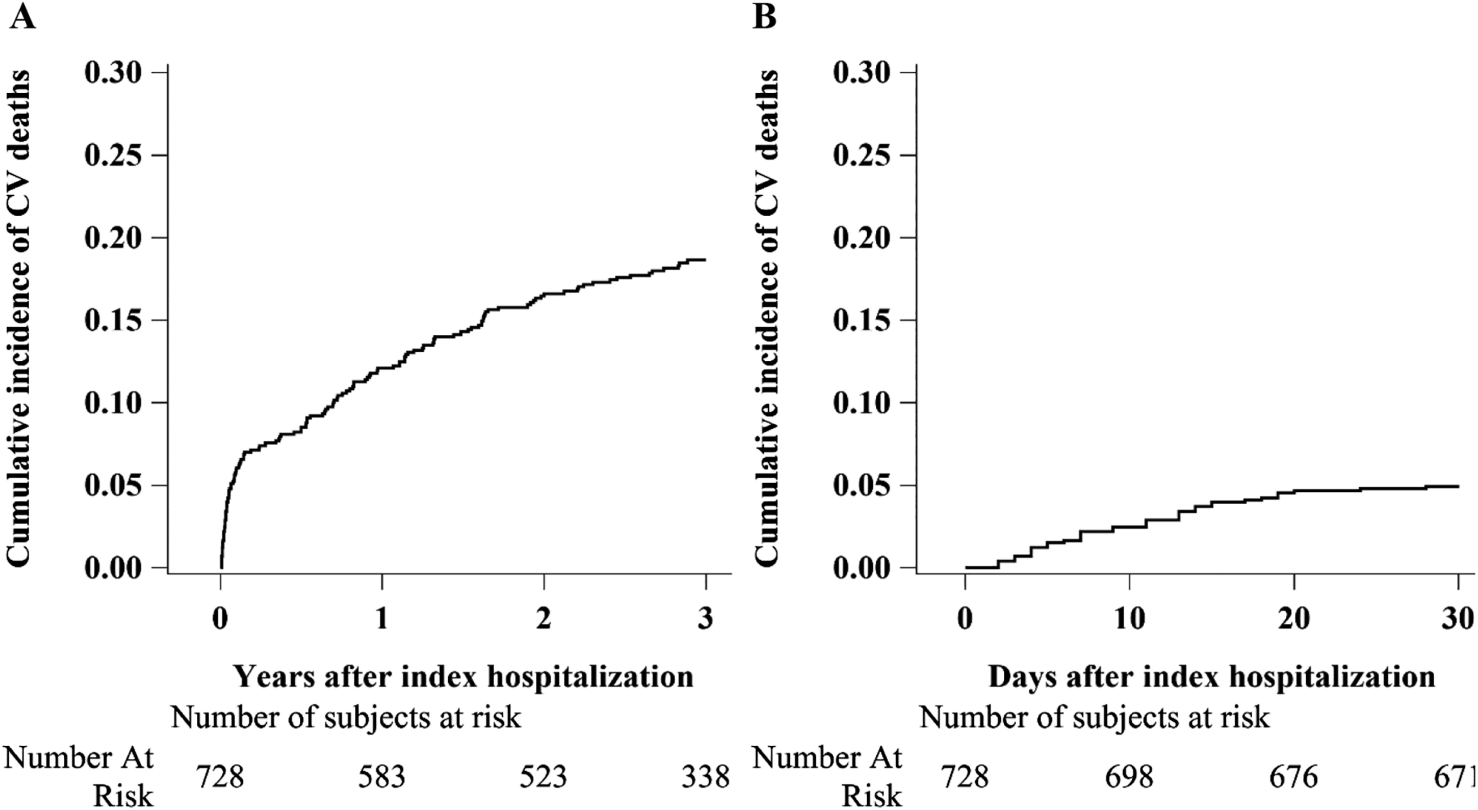
Cumulative incidence functions for cardiovascular (CV) death, considering death for other causes as a competing event, during the entire follow‐up after index hospitalization (A) and during index hospitalization time (B).

Kaplan–Meier survival curves are shown in *Figure* *2*, and cumulative incidence functions in *Figure* *3*. Estimated overall 1 and 3 year mortality were, respectively, 0.20 (95% CI: 0.17–0.23) and 0.33 (95% CI: 0.30–0.37), and cumulative incidence of CV deaths after 1 and 3 years were, respectively, 0.12 (95% CI: 0.10–0.15) and 0.19 (95% CI: 0.16–0.22). In‐hospital mortality estimate after 30 days was 0.08 (95% CI: 0.06–0.10), and cumulative incidence of CV deaths was 0.05 (0.04–0.07).

### Multivariable analysis

We investigated which variables were associated with at least one of the four endpoints. The results are shown in *Table* *1*, for mortality during the entire follow‐up, and in *Table* *2*, for in‐hospital endpoints. Several covariates including age, NYHA class, SBP, non‐cardiac co‐morbidities, oxygen saturation, haemoglobin, serum creatinine, serum sodium, potassium, TnI, and EF were retained in the Cox and Fine and Gray analyses (*Tables* *3* and *4*). One hundred forty‐one (19%) patients were excluded from the final models due to missing values in at least one covariate. Despite their clinical relevance and statistical significance, concentration of NT‐proBNP and of CRP were excluded from the analyses due to a lack of values in more than 20% of patients.

**Table 1:** Patients characteristics by strata of all‐cause death status and of cardiovascular death status, during the entire follow‐up after index hospitalization (*N* = 728)

	Survivors *N* = 472	All‐cause deaths *N* = 256	*P*	CV survivors *N* = 588	CV deaths *N* = 140	*P*
Index hospitalization, *N* (%)						
ED admission time						
Night‐time (8 pm–8 am)	111 (23.5)	70 (27.3)	0.29	143 (24.3)	38 (27.1)	0.56
Daytime (8 am–8 pm)	361 (76.5)	186 (72.7)		445 (75.7)	102 (72.9)	
Department						
Cardiology/CICU	162 (34.3)	68 (26.6)	0.04	189 (32.1)	41 (29.3)	0.51
Medicine	285 (60.4)	174 (68.0)		366 (62.2)	93 (66.4)	
Clinical presentation, median (IQR)						
Gender, *N* (%)						
M	264 (55.9)	154 (60.2)	0.31	331 (56.3)	87 (62.1)	0.25
F	208 (44.1)	102 (39.8)		257 (43.7)	53 (37.9)	
Age	81 (74–86)	84 (77–89)	<0.001	81 (75–87)	84 (77–87)	0.04
SBP (mmHg)	130 (115–150)	125 (105–150)	0.004	130 (115–150)	120 (104–145)	0.004
DBP (mmHg)	70 (60–82)	70 (60–80)	0.001	70 (60–80)	68 (60–80)	0.02
HR	80 (66–95)	80 (65–91)	0.56	80 (66–94)	80 (67–93)	0.58
Oxygen saturation (%)	95 (92–98)	94 (90–97)	0.002	95 (92–97)	94 (89–97)	0.04
NYHA class, *N* (%)						
I–II–III	354 (75.0)	159 (62.1)	<0.001	429 (73.0)	84 (60.0)	0.003
IV	116 (24.6)	96 (37.5)		157 (26.7)	55 (39.3)	
Medical history, *N* (%)						
AMI	178 (37.7)	116 (45.3)	0.06	227 (38.6)	67 (47.9)	0.06
Atrial fibrillation	266 (56.4)	157 (61.3)	0.24	336 (57.1)	87 (62.1)	0.31
PM/ICD	143 (30.3)	95 (37.1)	0.07	179 (30.4)	59 (42.1)	0.01
CABG	52 (11.0)	37 (14.5)	0.22	66 (11.2)	23 (16.4)	0.12
Non‐cardiac co‐morbidities						
0	83 (17.6)	29 (11.3)	<0.001	96 (16.3)	16 (11.4)	0.12
1	145 (30.7)	74 (28.9)		179 (30.4)	40 (28.6)	
2	144 (30.5)	64 (25.0)		172 (29.3)	36 (25.7)	
≥3	95 (20.1)	88 (34.4)		136 (23.1)	47 (33.4)	
Biochemical profile and echocardiography, median (IQR)						
Hb (g/dL)	11.7 (10.4–12.9)	11.3 (10.3–12.6)	0.02	11.7 (10.5–12.8)	11.1 (10.0–12.7)	0.03
Ht (%)	36.4 (32.4–39.8)	35.0 (32.2–39.0)	0.05	36.2 (32.5–39.5)	34.6 (31.2–39.2)	0.07
WBC (×10^9^/L)	8.3 (6.4–10.7)	8.7 (6.6–11.6)	0.31	8.4 (6.4–10.8)	8.9 (6.7–11.9)	0.20
CRP (mg/L)^a^	11.4 (4.1–40.6)	20.1 (8.8–50.3)	<0.001	12.6 (4.8–43.1)	19.2 (8.2–50.2)	0.01
Serum creatinine (mg/dL)	1.14 (0.87–1.61)	1.51 (1.08–2.28)	<0.001	1.19 (0.89–1.70)	1.63 (1.16–2.37)	<0.001
Bilirubin (mg/dL)	0.60 (0.42–0.86)	0.60 (0.45–0.92)	0.46	0.60 (0.41–0.86)	0.63 (0.50–0.98)	0.07
Serum Na (mmol/L)	139 (136–141)	139 (136–141)	0.07	139 (136–141)	139 (136–141)	0.36
K (mmol/L)	4.0 (3.7–4.4)	4.2 (3.8–4.5)	0.001	4.0 (3.7–4.4)	4.2 (3.9–4.5)	0.01
AST (U/L)^a^	21 (16–31)	24 (15–33)	0.34	21 (16–31)	24 (14–37)	0.30
ALT (U/L)	25 (19–36)	23 (16–34)	0.06	25 (18–35)	24 (16–37)	0.84
TnI (ng/L)	0.03 (0.01–0.07)	0.05 (0.02–0.11)	<0.001	0.03 (0.01–0.07)	0.05 (0.02–0.11)	<0.001
NT‐proBNP (ng/L)^a^	3984 (2035–8144)	6537 (3098–14 429)	<0.001	4266 (2085–9132)	7219 (3649–14 370)	<0.001
EF (%), *N* (%)						
<40	164 (34.7)	127 (49.6)	0.001	217 (36.9)	74 (52.9)	0.002
40–49	111 (23.5)	48 (18.8)		133 (22.6)	26 (18.6)	
≥50	192 (40.7)	80 (31.2)		233 (39.6)	39 (27.9)	
IV therapy and other procedures, *N* (%)						
Furosemide	407 (86.2)	231 (90.2)	0.08	511 (86.9)	127 (90.7)	0.14
Inotropes	21 (4.4)	77 (30.1)	<0.001	47 (8.0)	51 (36.4)	<0.001
Vasodilators	109 (23.1)	51 (19.9)	0.36	129 (21.9)	31 (22.1)	1.00
PTCA	7 (1.5)	3 (1.2)	1.00	8 (1.4)	2 (1.4)	1.00
CVVH	8 (1.7)	24 (9.4)	<0.001	18 (3.1)	14 (10.0)	0.001

ALT, alanine transaminase; AMI, acute myocardial infarction; AST, aspartate transaminase; CABG, coronary artery bypass graft; CICU, Cardiac Intensive Care Unit; CRP, C‐reactive protein; CV, cardiovascular; CVVH, continuous venovenous haemofiltration; DBP, diastolic blood pressure; ED, emergency department; EF, ejection fraction; F, female; Hb, haemoglobin; HR, heart rate; Ht, haematocrit; ICD, implantable cardioverter‐defibrillator; IQR, interquartile range; IV, intravenous; K, potassium; M, male; Na, sodium; NT‐proBNP, N‐terminal pro‐brain natriuretic peptide; NYHA, New York Heart Association; PM, pace‐maker; PTCA, percutaneous transluminal coronary angioplasty; SBP, systolic blood pressure; TnI, Troponin I; WBC, white blood cell.

Non‐cardiac co‐morbidities: diabetes, dyslipidaemia, depression, Alzheimer's disease, renal insufficiency, chronic obstructive pulmonary disease, and stroke/transient ischaemic attack.

^a^ More than 20% of missing values.

^b^ More than 50% of missing values.

**Table 2:** Patients characteristics by strata of all‐cause death status and of cardiovascular death status, during index hospitalization time (*N* = 728)

	Survivors *N* = 665	Deaths *N* = 63	*P*	CV survivors *N* = 686	CV deaths *N* = 42	*P*
Index hospitalization, *N* (%)						
ED admission time						
Night‐time (8 pm–8 am)	162 (24.4)	19 (30.2)	0.39	167 (24.3)	14 (33.3)	0.26
Daytime (8 am–8 pm)	503 (75.6)	44 (69.8)		519 (75.7)	28 (66.7)	
Department						
Cardiology/CICU	210 (31.6)	20 (31.7)	1.00	215 (31.3)	15 (35.7)	0.78
Medicine	418 (62.9)	41 (65.1)		433 (63.1)	26 (61.9)	
Clinical presentation, median (IQR)						
Gender, *N* (%)						
M	382 (57.4)	36 (57.1)	1.00	393 (57.3)	25 (59.5)	0.90
F	283 (42.6)	27 (42.9)		293 (42.7)	17 (40.5)	
Age	81 (75–87)	85 (77–89)	0.01	82 (75–87)	85 (76–88)	0.30
SBP (mmHg)	130 (115–150)	115 (95–145)	0.01	130 (110–150)	115 (100–140)	0.01
DBP (mmHg)	70 (60–80)	65 (55–80)	0.05	70 (60–80)	69 (55–80)	0.23
HR	80 (66–94)	77 (63–90)	0.21	80 (66–94)	80 (70–90)	0.78
Oxygen saturation (%)	95 (92–97)	93 (89–97)	0.003	95 (91–97)	93 (89–97)	0.06
NYHA class, *N* (%)						
I–II–III	488 (73.4)	25 (39.7)	<0.001	494 (72.0)	19 (45.2)	<0.001
IV	174 (26.2)	38 (60.3)		189 (27.6)	23 (54.8)	
Medical history, *N* (%)						
AMI	266 (40.0)	28 (44.4)	0.59	274 (39.9)	20 (47.6)	0.42
Atrial fibrillation	381 (57.3)	42 (66.7)	0.16	395 (57.6)	28 (66.7)	0.25
PM/ICD	212 (31.9)	26 (41.3)	0.17	218 (31.8)	20 (47.6)	0.05
CABG	83 (12.5)	6 (9.5)	0.65	85 (12.4)	4 (9.5)	0.79
Non‐cardiac co‐morbidities						
0	109 (16.4)	3 (4.8)	0.09	110 (16.0)	2 (4.8)	0.10
1	197 (29.6)	22 (34.9)		204 (29.7)	15 (35.7)	
2	192 (28.9)	16 (25.4)		199 (29.0)	9 (21.4)	
≥3	162 (24.4)	21 (33.3)		168 (24.49)	15 (35.7)	
Biochemical profile and echocardiography, median (IQR)						
Hb (g/dL)	11.6 (10.4–12.8)	11.2 (10.1–12.8)	0.47	11.6 (10.4–12.8)	11.1 (10.1–12.9)	0.59
Ht (%)	36.0 (32.4–39.4)	34.7 (31.5–40.0)	0.66	35.9 (32.4–39.4)	34.2 (31.4–40.7)	0.75
WBC (×10^9^/L)	8.4 (6.4–10.7)	9.7 (7.5–13.4)	0.01	8.4 (6.4–10.8)	9.7 (7.4–13.4)	0.03
CRP (mg/L)^a^	12.1 (4.9–40.6)	37.0 (14.4–100.0)	<0.001	12.5 (5.1–43.0)	36.4 (14.4–101.0)	<0.001
Serum creatinine (mg/dL)	1.23 (0.92–1.74)	1.96 (1.23–2.78)	<0.001	1.23 (0.92–1.77)	1.98 (1.39–2.71)	<0.001
Bilirubin (mg/dL)	0.60 (0.42–0.86)	0.65 (0.52–0.98)	0.08	0.60 (0.42–0.86)	0.63 (0.55–1.01)	0.10
Serum Na (mmol/L)	139 (136–141)	137 (133–139)	<0.001	139 (136–141)	137 (134–139)	0.01
K (mmol/L)	4.0 (3.7–4.4)	4.2 (3.7–4.7)	0.14	4.0 (3.7–4.4)	4.2 (3.6–4.6)	0.79
AST (U/L)^a^	22 (16–31)	25 (14–42)	0.11	22 (16–31)	20 (13–45)	1.00
ALT (U/L)	25 (18–36)	25 (16–32)	0.72	25 (18–36)	24 (16–32)	0.50
TnI (ng/L)	0.03 (0.01–0.07)	0.09 (0.04–0.19)	<0.001	0.03 (0.01–0.08)	0.09 (0.04–0.17)	<0.001
NT‐proBNP (ng/L)^a^	4452 (2142–9226)	11 749 (6374–20 213)	<0.001	4629 (2172–9910)	11 020 (3643–17 405)	0.01
EF (%), *N* (%)						
<40	248 (37.3)	43 (68.3)	<0.001	259 (37.8)	32 (76.2)	<0.001
40–49	149 (22.4)	10 (15.9)		154 (22.4)	5 (11.9)	
≥50	262 (39.4)	10 (15.9)		267 (38.9)	5 (11.9)	
IV therapy and other procedures, *N* (%)						
Furosemide	583 (87.7)	55 (87.3)	0.72	600 (87.5)	38 (90.5)	0.37
Inotropes	60 (9.0)	38 (60.3)	<0.001	72 (10.5)	26 (61.9)	<0.001
Vasodilators	143 (21.5)	17 (27.0)	0.41	149 (21.7)	11 (26.2)	0.63
PTCA	8 (1.2)	2 (3.2)	0.41	9 (1.3)	1 (2.4)	1.00
CVVH	18 (2.7)	14 (22.2)	<0.001	22 (3.2)	10 (23.8)	<0.001

ALT, alanine transaminase; AMI, acute myocardial infarction; AST, aspartate transaminase; CABG, coronary artery bypass graft; CICU, Cardiac Intensive Care Unit; CRP, C‐reactive protein; CV, cardiovascular; CVVH, continuous venovenous haemofiltration; DBP, diastolic blood pressure; ED, emergency department; EF, ejection fraction; F, female; Hb, haemoglobin; HR, heart rate; Ht, haematocrit; ICD, implantable cardioverter‐defibrillator; IQR, interquartile range; IV, intravenous; K, potassium; M, male; Na, sodium; NT‐proBNP, N‐terminal pro‐brain natriuretic peptide; NYHA, New York Heart Association; PM, pace‐maker; PTCA, percutaneous transluminal coronary angioplasty; SBP, systolic blood pressure; TnI, Troponin I; WBC, white blood cell.

Non‐cardiac co‐morbidities: diabetes, dyslipidaemia, depression, Alzheimer's disease, renal insufficiency, chronic obstructive pulmonary disease, and stroke/transient ischaemic attack.

^a^ More than 20% of missing values.

^b^ More than 50% of missing values.

**Table 3:** Multiple Cox regression models for all‐cause death, during the entire follow‐up after index hospitalization (A) and during index hospitalization time (B), based on *N* = 587 patients

Parameter	(A) All‐cause deaths		(B) All‐cause in‐hospital deaths	
	HR (95% CI)	*P*	HR (95% CI)	*P*
Age (years)	1.04 (1.02–1.06)	<0.001	1.04 (1.00–1.08)	0.05
NYHA class				
IV vs. I‐II‐III	1.66 (1.22–2.26)	0.001	3.28 (1.71–6.27)	<0.001
SBP (mmHg)	0.99 (0.98–1.00)	<0.001	1.00 (0.99–1.01)	0.62
Non‐cardiac co‐morbidities				
1 vs. 0	1.21 (0.73–2.01)	0.46	2.70 (0.75–9.72)	0.13
2 vs. 0	0.94 (0.55–1.60)	0.81	0.95 (0.25–3.67)	0.94
≥3 vs. 0	1.65 (1.00–2.74)	0.05	1.34 (0.37–4.88)	0.66
Oxygen saturation (%)	0.97 (0.95–1.00)	0.03	0.95 (0.91–1.00)	0.07
Hb (g/dL)	0.97 (0.89–1.06)	0.50	0.97 (0.81–1.17)	0.78
Serum creatinine (mg/dL)	1.20 (1.09–1.32)	<0.001	1.22 (1.05–1.41)	0.01
Serum Na (mmol/L)	0.99 (0.96–1.02)	0.47	0.95 (0.9–1.01)	0.08
K (mmol/L)	0.98 (0.94–1.03)	0.46	0.98 (0.84–1.14)	0.78
TnI (ng/L)	0.93 (0.77–1.13)	0.47	0.97 (0.79–1.18)	0.76
EF (%)				
40–49 vs. <40	0.58 (0.39–0.84)	0.004	0.39 (0.18–0.86)	0.02
≥50 vs. <40	0.60 (0.43–0.84)	0.003	0.19 (0.07–0.47)	<0.001

CI, confidence interval; EF, ejection fraction; Hb, haemoglobin; HR, hazard ratio; K, potassium; Na, sodium; NYHA, New York Heart Association; SBP, systolic blood pressure; TnI, Troponin I.

One hundred forty‐one patients were excluded from the analyses due to missing values in at least one covariate.

**Table 4:** Multiple Fine and Gray regression models for cardiovascular death, considering death for other causes as a competing event, during the entire follow‐up after index hospitalization (A) and during index hospitalization time (B), based on *N* = 587 patients

Parameter	(A) CV deaths		(B) In‐hospital CV deaths	
	HR (95% CI)	*P*	HR (95% CI)	*P*
Age (years)	1.02 (1.00–1.04)	0.03	1.02 (0.98–1.05)	0.31
NYHA class				
IV vs. I–II–III	1.44 (0.94–2.20)	0.10	2.43 (1.15–5.11)	0.02
SBP (mmHg)	0.99 (0.98–1.00)	0.01	1.00 (0.99–1.01)	0.53
Non‐cardiac co‐morbidities				
1 vs. 0	1.31 (0.67–2.59)	0.43	2.75 (0.73–10.36)	0.13
2 vs. 0	1.10 (0.54–2.23)	0.80	1.14 (0.26–5.05)	0.86
≥3 vs. 0	1.42 (0.71–2.86)	0.33	1.42 (0.35–5.73)	0.62
Oxygen saturation (%)	0.96 (0.93–1.00)	0.03	0.93 (0.87–1.00)	0.05
Hb (g/dL)	0.96 (0.86–1.08)	0.53	1.05 (0.86–1.28)	0.63
Serum creatinine (mg/dL)	1.17 (1.02–1.34)	0.02	1.19 (1.00–1.42)	0.04
Serum Na (mmol/L)	1.00 (0.95–1.04)	0.90	0.98 (0.90–1.06)	0.58
K (mmol/L)	0.99 (0.97–1.00)	0.14	0.97 (0.89–1.06)	0.50
TnI (ng/mL)	0.81 (0.62–1.05)	0.12	0.79 (0.59–1.06)	0.11
EF (%)				
40–49 vs. <40	0.57 (0.34–0.95)	0.03	0.32 (0.11–0.97)	0.04
≥50 vs. <40	0.51 (0.33–0.80)	0.004	0.13 (0.04–0.41)	<0.001

CI, confidence interval; CV, cardiovascular; EF, ejection fraction; Hb, haemoglobin; HR, hazard ratio; K, potassium; Na, sodium; NYHA, New York Heart Association; SBP, systolic blood pressure; TnI, Troponin I.

One hundred forty‐one patients were excluded from the analyses due to missing values in at least one covariate.

At multivariable analysis, age (HR for +5 years: 1.22, *P* < 0.001), NYHA class IV vs. I–II–III (HR: 1.66, *P* = 0.001), SBP (HR for +5 mmHg: 0.95, *P* < 0.001), non‐cardiac co‐morbidities (HR for ≥3 vs. 0: 1.65, *P* = 0.05), oxygen saturation (HR for +5%: 0.86, *P* = 0.03), serum creatinine (HR for +1 mg/dL: 1.20, *P* < 0.001), and EF (HR for 40–49% vs. <40%: 0.58, *P* = 0.004; HR for ≥50% vs. <40%: 0.60, *P* = 0.003) were independent predictors of all‐cause mortality during the entire follow‐up. Similar results were obtained for CV mortality.

When considering in‐hospital deaths, NYHA class IV vs. I–II–III (HR: 3.28, *P* < 0.001), serum creatinine (HR for +1 mg/dL: 1.22, *P* = 0.01), and EF (HR for 40–49% vs. <40%: 0.39, *P* = 0.02; HR for ≥50% vs. <40%: 0.19, *P* < 0.001) remained independent predictors.

When considering in‐hospital deaths for CV cause, serum creatinine (HR for +1 mg/dL: 1.19, *P* = 0.04) and EF (HR for 40–49% vs. <40%: 0.32, *P* = 0.04; HR for ≥50% vs. <40%: 0.13, 95% CI: 0.04–0.41, *P* < 0.001) remained independent predictors, together with NYHA class vs. I–II–III (HR: 2.43, *P* = 0.02) that returned to be a predictor.

### Sensitivity analysis

Given the great number of missing data, we performed two sensitivity analyses shown as Supporting Information. Firstly, we added NT‐proBNP and CRP to the final models described in the previous section, thus including potentially important predictors of the outcomes, but also allowing for fewer patients to be used. Secondly, we reproduced the final models described in the previous section by pooling the results of five multiple imputations to overcome the presence of missing data. In the first case, we found that CRP was statistically significantly associated with all‐cause deaths during the entire follow‐up, NT‐proBNP was associated with in‐hospital CV deaths, while the other predictors showed similar significance with HRs going in the same direction as in our final models (Supporting Information, *Tables* *S2* and *S3*). In the second case, the results we found through multiple imputation did not change the results already found with the complete case scenario (Supporting Information, *Tables* *S4* and *S5*).

## Discussion

This is a retrospective analysis focused on the impact of hospitalization for AHF on long‐term clinical outcomes and potential predictors of these in a real‐life population of ACF patients admitted to the ED with acute shortness of breath.

In this analysis, we have not included the effect of medical therapy on the outcome, although data from registries and clinical trials have repeatedly shown the beneficial impact of physicians' adherence to HF medication and dose optimization on mortality in patients with reduced EF.^6^, ^7^, ^8^ The QUALIFY international registry conducted on 6112 patients previously hospitalized for HF showed that adherence to guideline recommendations for angiotensin‐converting enzyme inhibitors, sartans, beta‐blockers, mineralocorticoid receptor antagonists, and ivabradine was associated with better prognosis in patients with >50% of recommended dosages at baseline.^9^

Previous and recent studies have largely described the relationship between clinical, biochemical, and echocardiographic parameters and mortality in patients admitted with AHF, and our results contribute to reinforce and extend these.^1^, ^5^

Contrary to the literature, we found more daytime hospitalization for AHF than night‐time. We did not find significant gender differences in terms of mortality.

The median age of our cohort was 82 years. Compared with the largest AHF registries, our population was about 10 years older (ADHERE: 73 years^4^ and ATTEND: 72 years^10^), but in line with another retrospective Italian analyses made by Castello *et al*.^11^ This finding is likely due to the population aging over the last 10 years and due to a greater control of predisposing factors for HF. We have also to consider that Italy has one of the oldest population in Europe. As expected and widely established, this analysis has identified age as an independent predictor for in‐hospital and long‐term mortality.

### Mortality rate

The all‐cause mortality rate was higher in patients hospitalized in the medicine department (*P* = 0.004). This is supposed to be related to the different characteristics of patients admitted. Generally, in the medicine department, patients are older and affected by several co‐morbidities compared with patients hospitalized in the cardiology unit. However, the overall CV mortality was similar in the two groups.

The 1 year crude mortality rate in the entire cohort was 20%, with the highest risk of death during the index hospitalization (with 8% estimate in‐hospital mortality at 30 days). A higher risk of events during the index hospitalization was more evident for the CV deaths, for which we found a cumulative 1 year incidence of 12% with a cumulative incidence in the first 30 days of hospitalization of about 5%.

Comparing our data with the ADHERE registry, in‐hospital mortality rate seems to be slightly higher. Despite this, the 1 year crude mortality rate was similar or lower than several analyses on the AHF registry.^4^, ^5^, ^12^, ^13^, ^14^ About it, the ESC‐HF long‐term registry was a recent prospective, observational study of 6629 AHF patients enrolled from April 2011 to June 2015 in 21 European countries.^15^ In the ESC‐HF long‐term registry, 1 year mortality rate was 26.7%, comparable with our results.

### Predictors of outcomes

At the multivariable analysis, we found for our cohort six independent predictors of long‐term all‐cause mortality: age, NYHA IV, SBP, serum creatinine, serum sodium, and EF. The EF was the only independent predictor of long‐term CV mortality too. As explained before, we could not use NT‐proBNP levels at baseline for the multivariable analysis due to the high percentage of missing data regarding this factor.

It is not surprising that NYHA class has several enforcements in patients with HF (diagnosis, decision‐making for therapy titration or devices implantation) and also that a patient admitted in hospital with NYHA class IV has a poor in‐hospital prognosis. An analysis performed using 4786 of the 4842 patients enrolled in the ATTEND registry showed that the in‐hospital mortality rate was significantly higher in patients admitted with NYHA IV and that the relationship between NYHA IV and mortality was stronger in patients with >75 years, in female patients, and in those with preserved EF.^16^ Our results support that NYHA class IV strongly predicts in‐hospital mortality but also add that NYHA IV at the index hospitalization is a predictor of all‐cause death even at long term.

Systolic blood pressure at admission turned out to be a clinical independent predictor of mortality, at both short and long terms. Lower SBP was associated to higher long‐term all‐cause and CV mortality, as confirmed in several other studies.^15^, ^17^, ^18^ Gheorghiade *et al*. enrolled both HF with preserved EF and HF with reduced HF (HFrEF) patients and proved a significant correlation between SBP values <120 mmHg on admission and a poor prognosis, despite medical therapy.^19^ Shiraishi *et al*. have studied 6232 patients between 2010 and 2012 admitted in the emergency room for AHF and showed that SBP at admission to the ED was inversely associated with in‐hospital death.^20^ There may be different effective explanations of this correlation. At first, hypotension makes the HF treatment limited when it comes to administration of angiotensin receptor–neprilysin inhibitors, angiotensin‐converting enzyme inhibitors, angiotensin receptor blockers, and beta‐blockers: at discharge, such patients may not have an optimized therapy, leading to a worse long‐term outcome. Furthermore, a low SBP is usually a sign of end‐stage HF. It is associated with a lower cardiac output and consequent cardiac ischaemia, peripheral hypoperfusion, worsening renal function, or multiorgan failure. Finally, the management of these patients often requires the use of inotropes due to a poor response to standard treatment. About this, there is a large consensus that the use of inotropes could increase the risk of adverse events and that they do not reduce the long‐term mortality.^21^, ^22^, ^23^ In our analysis, the use of inotropes and CVVH was related to poor both in‐hospital and long‐term prognoses.

Contrary to blood pressure, we did not find any association between early and late mortality and heart rate at admission. Contrary to observational studies we have not found a correlation between high heart rate values (> 70 bpm) and the increase in hospital mortality and all cause mortality.^24^, ^25^, ^26^ In our study, there was also no difference in terms of early and late mortality in patients admitted with atrial fibrillation compared with those admitted in sinus rhythm. The admission heart rate could be influenced by transitory neurohormonal activation that represents an adaptive mechanism that does not affect the medium–long‐term prognosis.^27^

Regarding the biochemical panel, high creatinine level at the time of admission was an independent predictor for both in‐hospital and long‐term all‐cause mortality (*P* = 0.01 and *P* < 0.001, respectively). The impact of creatinine and worsening renal failure on readmission and on in‐hospital, medium and long‐term mortality has been widely demonstrated in several studies.^28^, ^29^, ^30^ In a previous study similar to ours, Vaz Pérez *et al*. discovered that creatinine level at admission strongly predicts mortality at 1 and 5 years in 128 patients with AHF admitted in medicine department.^31^

Several studies showed a high prevalence of anaemia in these patients with AHF.^32^, ^33^, ^34^ In this study, patients hospitalized with AHF were anaemic (median 11.7 and 11.3 g/dL, respectively). Moreover, a low haemoglobin value is an independent factor of both mortality and morbidity and is related to a longer period of hospitalization.^35^ This is true for the admission value as much as for in‐hospital changes of haemoglobin concentration.^36^ In our study, blood concentration of haemoglobin is not an independent predictor factor of mortality (HR: 0.96, *P* = 0.53), but the univariate analysis showed a significant influence of haemoglobin for both all‐cause mortality and CV mortality during the entire follow‐up, in line with the current knowledge. The reason for the influence of haemoglobin on mortality has to be sought in the reduced oxygen‐carrying capacity of blood that brings to increased cardiac work, elevated demand‐related ischaemia, and renal hypoxia.^37^

Moreover, our multivariable analysis did not point high non‐sensitive TnI levels and hyponatremia as independent predictors of mortality, in contrast to several studies.^38^, ^39^, ^40^, ^41^, ^42^, ^43^

Finally, we found that an EF <40% was the only predictor of both all‐cause mortality and CV mortality at the multivariable analysis. In literature, the role of a low EF as a predictor of poor outcome in AHF patients is controversial. Some studies and meta‐analyses underline that the risk of mortality and rehospitalization for patients with HF and preserved or mid‐range EF is the same of those with reduced EF, but there are also a lot of studies that showed a lower rate of mortality in patients with EF >35%.^44^, ^45^ A recent meta‐analysis including 25 HF studies and 606 762 patients pointed out a significantly high mortality rate in HFrEF compared with HF with mid‐range EF/HF with preserved EF but no differences regarding the CV mortality.^46^ A real‐life study enrolling 830 patients hospitalized for AHF analysed the predictor of mortality in order to create a multiparametric prognostic tool.^47^ Similarly to what we reported in our observational analysis, they also experienced that a low EF was associated with mortality at 30 days, 6 months, and 5 years at multivariable analysis, as well as the mitral regurgitation and a creatinine >2 mg/dL.

### Limitations

The current study represents an analysis of a cohort of consecutive patients admitted for AHF. This study is a retrospective analysis and limited to a single hospital reality. Another limitation of the study is the relative small sample size that could lead to biases in patients' selection. Moreover, the biomarker levels, especially natriuretic peptides, were measured at baseline in a limited sample of patients. The diagnosis of HF based only on clinical signs and echocardiographic evidence of congestion should be considered less accurate compared when biomarkers are used to confirm the cardiac nature of dyspnoea. Finally, we have not analysed the effect of therapy, its variations, and adherence to contemporary pharmacologic treatment guidelines.^48^

This is the major limit of the study considering the importance and the effect on the short‐term and long‐term prognoses of adherence to recommended therapy especially in patients with HFrEF.^49^

## Conclusions

In this retrospective, single‐centre analysis, we found a similar mortality rate comparing with other analysis and with the most important registries. In patients admitted to the emergency department with acute shortness of breath due to AHF age,NYHA class IV, EF and baseline SBP, creatinine levels, and sodium levels were independent predictors of 1year mortality. The EF was the only predictor of CV mortality too. These results need to be interpreted with caution due to the relative limited number of patients and the single‐centre experience.

## Conflict of interest

The authors have no conflict of interest in regard to this manuscript.

## Funding

None.

## Supporting information


**Table S1.** Non‐cardiac comorbidities of the study population (N = 728).
**Table S2.** Multiple Cox regression models for all‐cause death, during the entire follow‐up after index hospitalization (A), and during index hospitalization time (B), based on N = 312 patients.
**Table S3.** Multiple Fine and Gray regression models for CV death, considering death for other causes as a competing event, during the entire follow‐up after index hospitalization (A) and during index hospitalization time (B), based on N = 312 patients.
**Table S4.** Multiple Cox regression models for all‐cause death, during the entire follow‐up after index hospitalization (A), and during index hospitalization time (B), based on all N = 728 patients.
**Table S5.** Multiple Fine and Gray regression models for CV death, considering death for other causes as a competing event, during the entire follow‐up after index hospitalization (A) and during index hospitalization time (B) based on all N = 728 patients.
