# Supplementary material for: In‐hospital and long‐term mortality for acute heart failure: analysis at the time of admission to the emergency department
Source: ESC Heart Fail. 2020 Jun 26;7(5):2650–61. doi: 10.1002/ehf2.12847 (PMC7524058; doi:10.1002/ehf2.12847)
Supplement: Supplementary file 1 — Table S1. Non‐cardiac comorbidities of the study population (N = 728). Table S2. Multiple Cox regression models for all‐cause death, during the entire follow‐up after index hospitalization (A), and during index hospitalization time (B), based on N = 312 patients. Table S3. Multiple Fine and Gray regression models for CV death, considering death for other causes as a competing event, during the entire follow‐up after index hospitalization (A) and during index hospitalization time (B), based on N = 312 patients. Table S4. Multiple Cox regression models for all‐cause death, during the entire follow‐up after index hospitalization (A), and during index hospitalization time (B), based on all N = 728 patients. Table S5. Multiple Fine and Gray regression models for CV death, considering death for other causes as a competing event, during the entire follow‐up after index hospitalization (A) and during index hospitalization time (B) based on all N = 728 patients. [file EHF2-7-2650-s001.docx]

**SUPPLEMENTARY MATERIAL:**

**Supplementary Table 1. Non-cardiac comorbidities of the study population (N=728).**

| **Diabetes, N (%)** | 278 (38.2) |
| --- | --- |
| **Dyslipidaemia, N (%)** | 176 (24.2) |
| **Depression, N (%)** | 42 (5.8) |
| **Alzheimer’s disease, N (%)** | 21 (2.9) |
| **Renal insufficiency, N (%)** | 386 (53.0) |
| **COPD, N (%)** | 233 (32.0) |
| **Stroke/TIA, N (%)** | 107 (14.7) |

COPD: chronic obstructive pulmonary disease; TIA, transient ischemic attack

**Supplementary Table 2. Multiple Cox regression models for all-cause death, during the entire follow-up after index hospitalization (A), and during index hospitalization time (B), based on N=312 patients.**

|  |  | **(A) All-cause deaths** | | **(B) All-cause in-hospital deaths** | |
| --- | --- | --- | --- | --- | --- |
| **Parameter** |  | **HR (95% CI)** | ***p*** | **HR (95% CI)** | ***p*** |
| **Age, years** |  | 1.04 (1.01-1.06) | *0.01* | 1.08 (1.01-1.16) | *0.03* |
| **NYHA class** | **IV vs. I-II-III** | 1.52 (0.97-2.36) | *0.06* | 5.22 (1.94-14.08) | *0.001* |
| **SBP, mmHg** |  | 0.99 (0.98-1.00) | *0.05* | 0.99 (0.97-1.00) | *0.15* |
| **Non cardiac comorbidities** | **1 vs. 0** | 1.32 (0.65-2.71) | *0.44* | 2.62 (0.51-13.35) | *0.25* |
|  | **2 vs. 0** | 1.10 (0.52-2.31) | *0.81* | 0.85 (0.13-5.51) | *0.86* |
|  | **≥3 vs. 0** | 2.19 (1.09-4.40) | *0.03* | 0.90 (0.15-5.24) | *0.90* |
| **Oxygen saturation, %** |  | 0.98 (0.94-1.02) | *0.30* | 0.95 (0.87-1.04) | *0.28* |
| **Hb, g/dL** |  | 0.98 (0.87-1.10) | *0.72* | 1.08 (0.78-1.50) | *0.63* |
| **CRP, mg/L** |  | 1.01 (1.00-1.01) | *<0.001* | 1.01 (1.00-1.01) | *0.10* |
| **Serum creatinine, mg/dL** |  | 1.23 (1.02-1.49) | *0.03* | 1.76 (1.12-2.77) | *0.02* |
| **Serum Na, mmol/L** |  | 1.00 (0.96-1.05) | *0.93* | 1.07 (0.96-1.20) | *0.21* |
| **K, mmol/L** |  | 0.96 (0.87-1.06) | *0.42* | 1.00 (0.85-1.17) | *0.96* |
| **TnI, ng/L** |  | 0.83 (0.64-1.08) | *0.17* | 0.93 (0.74-1.17) | *0.54* |
| **NT-proBNP, ng/L** |  | 1.00 (1.00-1.00) | *0.21* | 1.00 (1.00-1.00) | *0.08* |
| **EF, %** | **40-49 vs. <40** | 0.64 (0.39-1.05) | *0.08* | 0.60 (0.21-1.70) | *0.34* |
|  | **≥50 vs. <40** | 0.66 (0.41-1.05) | *0.08* | 0.20 (0.05-0.73) | *0.02* |

*416 patients were excluded from the analyses due to missing values in at least one covariate.*

**Supplementary Table 3. Multiple Fine and Gray regression models for CV death, considering death for other causes as a competing event, during the entire follow-up after index hospitalization (A) and during index hospitalization time (B), based on N=312 patients.**

|  |  | **(A) CV deaths** | | **(B) In-hospital CV deaths** | |
| --- | --- | --- | --- | --- | --- |
| **Parameter** |  | **HR (95% CI)** | ***p*** | **HR (95% CI)** | ***p*** |
| **Age, years** |  | 1.01 (0.98-1.04) | *0.42* | 1.03 (0.98-1.09) | *0.21* |
| **NYHA class** | **IV vs. I-II-III** | 2.24 (1.12-4.46) | *0.02* | 8.40 (1.66-42.39) | *0.01* |
| **SBP, mmHg** |  | 1.00 (0.99-1.01) | *0.39* | 1.00 (0.99-1.02) | *0.72* |
| **Non cardiac comorbidities** | **1 vs. 0** | 1.25 (0.46-3.41) | *0.66* | 2.62 (0.34-19.88) | *0.35* |
|  | **2 vs. 0** | 0.77 (0.28-2.16) | *0.62* | 0.24 (0.02-2.44) | *0.23* |
|  | **≥3 vs. 0** | 1.30 (0.48-3.54) | *0.61* | 1.11 (0.16-7.67) | *0.92* |
| **Oxygen saturation, %** |  | 1.01 (0.94-1.09) | *0.8* | 0.92 (0.82-1.04) | *0.17* |
| **Hb, g/dL** |  | 0.93 (0.77-1.12) | *0.44* | 1.24 (0.77-1.98) | *0.38* |
| **CRP, mg/L** |  | 1.01 (1.00-1.01) | *0.004* | 1.00 (1.00-1.01) | *0.84* |
| **Serum creatinine, mg/dL** |  | 1.35 (1.10-1.66) | *0.004* | 3.06 (1.90-4.93) | *<0.001* |
| **Serum Na, mmol/L** |  | 0.97 (0.91-1.02) | *0.22* | 1.10 (0.95-1.27) | *0.22* |
| **K, mmol/L** |  | 0.95 (0.88-1.03) | *0.21* | 0.96 (0.78-1.19) | *0.72* |
| **TnI, ng/mL** |  | 0.72 (0.41-1.28) | *0.27* | 0.02 (0.00-4.15) | *0.15* |
| **NT-proBNP, ng/L** |  | 1.00 (1.00-1.00) | *0.09* | 1.00 (1.00-1.00) | *0.01* |
| **EF, %** | **40-49 vs. <40** | 0.50 (0.24-1.02) | *0.06* | 0.33 (0.08-1.35) | *0.12* |
|  | **≥50 vs. <40** | 0.38 (0.19-0.76) | *0.01* | 0.11 (0.03-0.49) | *0.004* |

*416 patients were excluded from the analyses due to missing values in at least one covariate.*

**Supplementary Table 4. Multiple Cox regression models for all-cause death, during the entire follow-up after index hospitalization (A), and during index hospitalization time (B), based on all N=728 patients.**

|  |  | **(A) All-cause deaths** | | **(B) All-cause in-hospital deaths** | |
| --- | --- | --- | --- | --- | --- |
| **Parameter** |  | **HR (95% CI)** | ***p*** | **HR (95% CI)** | ***p*** |
| **Age, years** |  | 1.04 (1.03-1.06) | *<0.001* | 1.05 (1.02-1.09) | *0.01* |
| **NYHA class** | **IV vs. I-II-III** | 1.67 (1.27-2.19) | *<0.001* | 2.91 (1.66-5.10) | *<0.001* |
| **SBP, mmHg** |  | 0.99 (0.99-1.00) | *<0.001* | 0.99 (0.99-1.00) | *0.25* |
| **Non cardiac comorbidities** | **1 vs. 0** | 1.29 (0.83-1.99) | *0.26* | 3.62 (1.06-12.35) | *0.04* |
|  | **2 vs. 0** | 1.01 (0.64-1.59) | *0.97* | 1.56 (0.43-5.60) | *0.50* |
|  | **≥3 vs. 0** | 1.76 (1.14-2.73) | *0.01* | 2.14 (0.61-7.50) | *0.23* |
| **Oxygen saturation, %** |  | 0.96 (0.94-0.99) | *0.001* | 0.96 (0.91-1.01) | *0.09* |
| **Hb, g/dL** |  | 0.98 (0.91-1.05) | *0.58* | 1.08 (0.93-1.25) | *0.32* |
| **Serum creatinine, mg/dL** |  | 1.17 (1.08-1.26) | *<0.001* | 1.20 (1.05-1.38) | *0.01* |
| **Serum Na, mmol/L** |  | 0.98 (0.96-1.01) | *0.22* | 0.94 (0.90-0.99) | *0.02* |
| **K, mmol/L** |  | 0.99 (0.97-1.01) | *0.38* | 0.97 (0.86-1.08) | *0.6* |
| **TnI, ng/L** |  | 0.98 (0.85-1.12) | *0.72* | 1.06 (0.93-1.21) | *0.39* |
| **EF, %** | **40-49 vs. <40** | 0.58 (0.41-0.81) | *0.001* | 0.43 (0.21-0.89) | *0.02* |
|  | **≥50 vs. <40** | 0.61 (0.46-0.83) | *0.001* | 0.28 (0.13-0.60) | *0.001* |

*Multiple imputation has been used for 141 patients who had missing values in at least one covariate. Results and t-test p-values from 5 imputations, each obtained using the fully conditional specification (FCS) method, with discriminant method for the imputation of the class variables, and regression method for continuous variables.*

**Supplementary Table 5. Multiple Fine and Gray regression models for CV death, considering death for other causes as a competing event, during the entire follow-up after index hospitalization (A) and during index hospitalization time (B) based on all N=728 patients.**

|  |  | **(A) CV deaths** | | **(B) In-hospital CV deaths** | |
| --- | --- | --- | --- | --- | --- |
| **Parameter** |  | **HR (95% CI)** | ***p*** | **HR (95% CI)** | ***p*** |
| **Age, years** |  | 1.03 (1.01-1.04) | *0.01* | 1.02 (0.99-1.06) | *0.19* |
| **NYHA class** | **IV vs. I-II-III** | 1.47 (1.02-2.13) | *0.04* | 2.06 (1.05-4.04) | *0.04* |
| **SBP, mmHg** |  | 0.99 (0.98-1.00) | *0.01* | 1.00 (0.99-1.01) | *0.46* |
| **Non cardiac comorbidities** | **1 vs. 0** | 1.15 (0.64-2.07) | *0.64* | 3.22 (0.82-12.58) | *0.09* |
|  | **2 vs. 0** | 1.01 (0.54-1.87) | *0.98* | 1.44 (0.33-6.36) | *0.63* |
|  | **≥3 vs. 0** | 1.43 (0.79-2.59) | *0.23* | 2.01 (0.47-8.61) | *0.35* |
| **Oxygen saturation, %** |  | 0.95 (0.92-0.99) | *0.01* | 0.96 (0.89-1.03) | *0.21* |
| **Hb, g/dL** |  | 0.97 (0.88-1.08) | *0.58* | 1.11 (0.93-1.32) | *0.25* |
| **Serum creatinine, mg/dL** |  | 1.15 (1.04-1.27) | *0.01* | 1.18 (1.02-1.37) | *0.03* |
| **Serum Na, mmol/L** |  | 1.00 (0.96-1.04) | *0.87* | 0.96 (0.90-1.03) | *0.27* |
| **K, mmol/L** |  | 0.99 (0.97-1.01) | *0.43* | 0.97 (0.82-1.14) | *0.67* |
| **TnI, ng/L** |  | 0.84 (0.64-1.10) | *0.21* | 0.95 (0.78-1.16) | *0.62* |
| **EF, %** | **40-49 vs. <40** | 0.60 (0.38-0.95) | *0.03* | 0.30 (0.11-0.84) | *0.02* |
|  | **≥50 vs. <40** | 0.55 (0.37-0.82) | *0.003* | 0.21 (0.08-0.53) | *0.001* |

*Multiple imputation has been used for 141 patients who had missing values in at least one covariate. Results and t-test p-values from 5 imputations, each obtained using the fully conditional specification (FCS) method, with discriminant method for the imputation of the class variables, and regression method for continuous variables.*
